# Assessing proteases and enzymes of the trypanothione system in subpopulations of *Leishmania (Viannia) braziliensis* Thor strain during macrophage infection

**DOI:** 10.1590/0074-02760240038

**Published:** 2024-07-08

**Authors:** Barbara Cristina de Albuquerque-Melo, Bernardo Acácio Santini Pereira, Vítor Ennes-Vidal, Maria Eduarda Pinto Gonçalves, Luzia Monteiro de Castro Côrtes, Léa Cysne-Finkelstein, Herbert Leonel de Matos Guedes, Geovane Dias-Lopes, Carlos Roberto Alves

**Affiliations:** 1 Fundação Oswaldo Cruz-Fiocruz, Instituto Oswaldo Cruz, Laboratório de Biologia Molecular e Doenças Endêmicas, Rio de Janeiro, RJ, Brasil; 2 Universidade Federal Fluminense, Faculdade de Medicina, Departamento de Patologia, Niterói, RJ, Brasil; 3 Fundação Oswaldo Cruz-Fiocruz, Instituto Oswaldo Cruz, Laboratório de Doenças Parasitárias, Rio de Janeiro, RJ, Brasil; 4 Fundação Oswaldo Cruz-Fiocruz, Instituto Oswaldo Cruz, Laboratório de Imunoparasitologia, Rio de Janeiro, RJ, Brasil; 5 Fundação Oswaldo Cruz-Fiocruz, Instituto Oswaldo Cruz, Laboratório de Imunologia Clínica, Rio de Janeiro, RJ, Brasil; 6 Universidade Federal do Rio de Janeiro, Instituto de Microbiologia Paulo de Góes, Laboratório de Imunobiotecnologia, Rio de Janeiro, RJ, Brasil

**Keywords:** Leishmania (Viannia) braziliensis, cysteine proteases, serine proteases, trypanothione system, virulence factors

## Abstract

**BACKGROUND:**

*Leishmania (Viannia) braziliensis* Thor strain exhibits a heterogeneous composition comprised of subpopulations with varying levels of infectivity. Clonal subpopulations were previously obtained from the strain Thor by sorting single-parasites and proceeding cultivation. The subpopulations used in this study are named Thor03, Thor 10 and Thor22.

**OBJECTIVES:**

Phenotypic characteristics of the parasite, specially focusing on virulence factors and resistance to the antimicrobial mechanisms of macrophages, were investigate in these subpopulations.

**METHODS:**

Cellular and molecular biology, as well as biochemistry approaches were applied to obtain the data analysed in this study.

**FINDINGS:**

Relative quantification of gene expression was measured for calpain, cysteine protease B (CPB), and subtilisin proteases but no significant differences in these genes’ expression among subpopulations was observed. However, subtilisin and CPB proteins were assessed as more abundant in Thor03 by fluorescence-labelled flow cytometry technique. Western Blotting assays, as semi-quantitative analysis in gel, showed higher concentrations of subtilisin (110 to 50 kDa) and CPB (40 to 18 kDa) in extract of intracellular amastigotes from subpopulations Thor03 and Thor10 and calpain (60 to 25 kDa) showed no significant differences among subpopulations. Complementary, higher trypanothione reductase activity was observed in Thor10 intracellular amastigotes and assays of susceptibility to hydrogen peroxide-inducing agents and nitric oxide donors conducted with promastigotes revealed greater resistance to *in vitro* oxidative stress induction for Thor10, followed by Thor03.

**MAIN CONCLUSIONS:**

The data obtained for the virulence factors explored here suggest how multiple coexisting phenotypic-distinct subpopulations may contribute in adaptability of a single *L. (V.) braziliensis* strain during infection in the host cells.

*Leishmania (Viannia) braziliensis* has been associated with a majority of American Tegumentary Leishmaniasis (ATL) cases in South America.^1^ ^,^ ^2^ This parasite is related with an array of distinct clinical forms, including localised cutaneous, mucosal, and disseminated leishmaniasis.^3^ ^,^ ^4^ The phenotypic plasticity of this species has been studied by exploring intrinsic factors of the field samples and performing *in vitro* assays,^5^ ^,^ ^6^ ^,^ ^7^ ^,^ ^8^ ^,^ ^9^ which have contributed to a better understanding of the disease and resistance to treatment.^10^ ^,^ ^11^

*Leishmania* spp., virulence factors influences this parasite infection dynamics.^12^ The action of the parasite proteolytic landscape is related to pathogenesis, performing functions in the host cell invasion and immune evasion.^13^ ^,^ ^14^ The composition of degradome of these parasites includes the cysteine proteases (CPs), which are well-known enzymes in *Leishmania* spp. due to their spectrum of action. One such CP, calpain plays a role in *Leishmania (Leishmania) amazonensis* amastigote intracellular development.^15^ The inhibition of calpains in *Leishmania* spp. reduced promastigote growth and infection rate in macrophage cells of the lineage RAW.^16^ The MDL28170 (Cbz-Val-Phe-H) is a potent peptide inhibitor of calpain, which promotes reduction on the parasite development in the mammalian cells, without presenting a cytotoxic effect on host cells.^16^ ^,^ ^17^ ^,^ ^18^ In addition, due to the previously described functions of these enzymes in the host-parasite relationship, their use as candidates for drug design has been proposed.^17^ ^,^ ^18^ ^,^ ^19^

Furthermore, the main characterised CP, the cathepsin L-like cysteine protease B (CPB), is found in megasomes of amastigotes,^20^ ^,^ ^21^ as well as on the surface of promastigote.^22^ ^,^ ^23^ CPB presents multiple isoforms, which are encoded by genes grouped in tandem, and it characteristically exhibits a C-terminal extension with ~ 100 amino acids.^21^ CPB-deficient mutants of *Leishmania (Leishmania) mexicana* remained with unchanged growth or differentiation, but their capacity to infect macrophages was severely reduced;^24^ also, they only elicited the development of discrete lesions in BALB/c mice.^25^ The comparison between strains of *L. (V.) braziliensis* showed a greater amount of CPB in the virulent strain as well as a decrease in the infectivity of this parasite in the presence of cysteine protease inhibitor.^26^ Furthermore, *L. (V.) braziliensis* parasites cultivated after successive passages presented a decrease in macrophage infection assays and in the amount of CPB proteins.^27^

Moreover, studies on serine proteases showed that Oligopeptidase B (OPB) expression is increased during *in vitro* infection by *L. (L.) amazonensis*.^28^ In addition, OPB-deficient mutants of *Leishmania (Leishmania) donovani* have decreased virulence in the BALB/c mice model, as well as, in *in vitro* assays.^29^ OPB enzyme was described as essential to modulate macrophages immune response, promoting alterations in the expression of host cell proteins involved in cytokine secretion, signal transduction, and the inflammatory response.^29^ The deletion of OPB gene in *Leishmania (Leishmania) major* has also a relevant impact, reducing the ability of parasites to infect and survive within macrophages in *in vitro* assays.^30^

Another well-known serine protease is the subtilisin (Clan SB, family S8), related to the C-terminal processing of peroxidases of the trypanothione system.^31^ The trypanosomatids presented a low mass thiol designated trypanothione (N1-N8-bis (glutathionyl) spermidine, T[S]_2_)^32^ The system based on trypanothione T[S]_2_ is formed by four enzymes: trypanothione synthetase (TS), trypanothione reductase (TR), tryparedoxin (TXN) and tryparedoxin peroxidase (TXNPx). This enzymatic system is essential for parasite survival during oxidative stress.^32^ ^,^ ^33^ ^,^ ^34^ Alterations in this system renders the parasites more susceptible to oxidative damage.^33^ ^,^ ^34^

Macrophages participate as a first line of defence against invading pathogens such as *Leishmania*.^35^ These cells have microbicidal mechanisms including reactive oxygen species (ROS) and reactive nitrogen species (RNS).^36^ Therefore, parasites whose survival and development are related to invading these cells must have an adaptation able to deal with such challenge. This approach was explored to assess the infectivity of promastigotes from subpopulations of *L. (V.) braziliensis* Thor strain, which were originated from single-cells separated by cell sorting.^37^ The analysis of phenotypic characteristics grouped these subpopulations into two clusters, being macrophage infectivity as the predominant characteristic.^37^ Subsequently, the combination of representatives of these clusters in the infection of murine macrophages was evaluated, highlighting the role of population structure in a host infection by this parasite.^38^

This study assesses virulence factors in *L. (V.) braziliensis* Thor strain subpopulations to advance in the search for proximate causes to these parasites, as well as the final effects on the host antimicrobial responses. The approach applied herein considers the analysis of the main cysteine and serine proteases associated with the infection process, as well as enzymes of the trypanothione system in promastigotes and intracellular amastigotes. The data gathered is unequivocal evidence that subpopulations of a same strain may present distinct infectivity profiles and different patterns of proteases expression.

## MATERIALS AND METHODS

*Chemicals and culture reagents* - Penicillin, Streptomycin, Schneider’s Drosophila, RPMI 1640, Brewer thioglycollate medium, Resazurin Sodium Salt, Alexa 488-labeled goat anti-rabbit IgG secondary antibody, and Horseradish peroxidase (HRP) conjugated secondary antibody (anti-rabbit-IgG-HRP antibody and anti-mouse-IgG-HRP antibody), Trypanothione trifluoroacetate salt (T[S]_2_) and Tert-butyl hydroperoxide solution were purchased from Sigma-Aldrich Chemical Co. (Missouri, USA). Foetal bovine serum (FBS), DTNB (Ellman’s Reagent) (5,5-dithio-bis-(2-nitrobenzoic acid) and Pierce™ BCA Protein Assay Kit, Power SYBR^®^ Green PCR Master Mix, TRIzol™ Reagent, SuperScript™ IV VILO™ Master Mix with ezDNase™, Maryland, USA), SuperSignal West Pico Chemiluminescent Substrate, Qubit ssDNA Assay Kit and Qubit™ Protein Assay Kit were obtained from Thermo Fisher Scientific (Massachusetts, USA). NADPH and Sodium nitrite were purchased from Merck Millipore (Massachusetts, USA). RNeasy Mini Kit was obtained from QIAGEN (Maryland, USA). The anti-calpain,^19^ anti-subtilisin^39^ and anti-cyspep^40^ primary antibodies used were previously developed and characterised. All reagents were of analytical grade or higher.

*Parasites* - Promastigotes of subpopulations Thor03, Thor10 and Thor22, which were previously isolated from the *L. (V.) braziliensis* Thor (MCAN/BR/1998/R619), were used.^37^ ^,^ ^38^ The promastigotes strain were cultivated in Schneider’s insect medium pH 7.2 supplemented with 20% FBS, 200 IU penicillin and 200 μg /mL streptomycin at 26ºC.^38^ The number of viable parasites was determined using the Neubauer Hemocytometer.

*Obtaining peritoneal macrophages and experimental infection protocol* - Macrophages were obtained from BALB/c mice (4- to 8-week-old females) by intraperitoneal injection of Brewer thioglycolate medium, as previously described.^38^ Then, RPMI 1640 medium was injected, and the peritoneal macrophages were aspirated using sterile syringes. Samples were centrifuged (520 × g, 5 min, 4ºC), seeded, at a density of 5.0 × 10^5^ /mL, in RPMI 1640 medium with 10% FBS in wells of 6-wells plates and incubated for 24 h (37ºC and 5% CO_2_). Later, stationary phase promastigotes (Thor03 and Thor10 at the 5th day of culture; Thor22 at the 4th day) were used in macrophage infection assays at a ratio of 10:1 (parasites: macrophages) with 5 h interaction (37ºC and 5% CO_2_).^38^ Following this period, the wells were washed with RPMI 1640 medium, and fresh medium containing 10% FBS was added. The total time of infection was 24 h, and non-infected macrophage cultures were used as control.

*Selection of sequences and primer design* - The primers used in this study were previously designed and used to detect cysteine and serine proteases, as well as trypanothione reductase (TR) and tryparedoxin peroxidase (TXNPx) transcripts [Supplementary data (Table I)]. The calpain genes (CALP1 - LbrM.18.1160; CALP2 - LbrM.20.0290; CALP3 - LbrM.20.5410; and CALP4 - LbrM.31.0600) showed higher expression in metacyclic than in procyclic promastigotes in a previous study with *L. (V.) braziliensis* Thor strain.^19^ The subtilisin was designated here as S13 (LbrM.13.0860), due to previous work that explored the two genes annotated as subtilisin in the genome of *L. (V.) braziliensis*.^8^ ^,^ ^9^ ^,^ ^41^ Among the enzymes of the trypanothione system, the TR primer is directed to the protein encoded by the LbrM.05.0350 gene; while TXNPx is targeted to the cytoplasmic isoform of the enzyme (LbrM.15.1080), which is associated with oxidative stress generated by external sources. Both housekeeping genes, 40S Ribosomal protein S8 (LbrM.24.2160) and Actin (LbrM.04.1250), were also previously designed and analysed for *L. (V.) braziliensis*.^8^ ^,^ ^9^ ^,^ ^19^ ^,^ ^42^ The primers designed for this study were based on the *L.* (*V.*) *braziliensis* CPB (LbrM.08.0810, LbrM.08.0820 and LbrM.08.0830) sequences recorded in the Genedb database (http://www.genedb.org). The primers were designed using the online software Primer-BLAST (https://www.ncbi.nlm.nih.gov/tools/primer-blast/index.cgi), with all parameters set to default except the product size range, which was adjusted to 80-250 base pairs (bp) [Supplementary data (Table I)]. All primers were synthesised by Invitrogen Brazil at a concentration of 25 nM and purified by desalting.

*RNA processing and reverse transcription quantitative polymerase chain reaction (RT-qPCR)* - Stationary-phase promastigotes (1.0 × 10^8^) and macrophages (5.0 × 10^6^) containing intracellular amastigotes were lysed in 1 mL TRIzol™. RNeasy Mini Kit was used to extract RNA from each sample, accordingly to the manufacturer’s protocol. RNA concentrations were assessed by spectrophotometry at 260/280 nm and 230/260 nm using NanoDrop 2000c spectrophotometer (ThermoScientific, Waltham, Massachusetts, USA). cDNA synthesis was performed using the SuperScript™ IV VILO™ Master Mix with ezDNase™ with a maximum of 2.5 µg of total RNA, as per manufacturer’s instructions, and cDNA concentrations were measured with Qubit ssDNA Assay Kit.

The RT-qPCR assays were performed in a 7500 Fast Real-Time PCR (Applied Biosystems, California, USA) using 2 μL cDNA per well (at 1 ng/μL) in a final reaction volume of 10 μL containing Power SYBR^®^ Green PCR Master Mix at 1× and forward and reverse primers at 0.3 µM. PCR cycling conditions comprised a first step at 95ºC for 10 min, followed by 40 cycles at 95ºC for 15 s and 56ºC for 1 min (annealing/extension phase). To check for the primer’s specificity, melting curves were generated after the 40 cycles. In the assays to assess calpain gene expression, a temperature of 60ºC was used for the annealing/extension phase. Gene expression was calculated by relative quantitation using the comparative Ct method (ΔΔCt), as previously described^43^ expressed as fold change (2^-ΔΔCt^) and showed as the relationship between the relative quantitation (RQ) of amastigotes divided by the value of promastigotes. Therefore, values greater than 1 mean greater expression in intracellular amastigotes.

*Flow cytometry* - Promastigotes of *L. (V.) braziliensis* (2.0 × 10^7^ parasites) were processed for flow cytometry as previously described.^19^ Briefly, parasites were fixed with 4% paraformaldehyde and permeabilised with 0.01 % Triton X-100. The cells were incubated at room temperature for 2 h with the anti-subtilisin polyclonal antibody (1:200) and anti-cyspep antibody (1:400).^39^ ^,^ ^40^ Then, the samples were washed three times with phosphate-buffered saline (PBS), then incubated during 1 h at room temperature with Alexa 488-labeled goat anti-rabbit IgG secondary antibody (1:500) for anti-subtilisin and anti-cyspep. The analyses were performed on a FACSCalibur flow cytometer equipped with a 15 mW argon laser emitting at 488 nm (BD Bioscience, California, USA). The omission of the primary antibody was used as a negative control. Each experimental population was first mapped using a two-parameter histogram of forward-angle light scatter versus side scatter. The mapped population (n = 10,000) was then analysed for log green fluorescence using a single parameter histogram, and the mean fluorescence intensity (MFI) of each experimental system was divided by the MFI from the auto-fluorescence controls to obtain the variation index.

*Western blotting* - Murine peritoneal macrophages infected with subpopulations Thor03, Thor10 or Thor22 and non-infected macrophage cultures were lysed with Galán buffer [10% glycerol, 0.6% Triton X-100, 100 mM Tris-HCl at pH 6.8, and 150 Mm NaCl]. The soluble proteins (crude extract) were recovered by centrifugation (20,000 g, 30 min, 4ºC) and stored at -20ºC. Protein concentrations were determined using Pierce™ BCA Protein Assay Kit. Crude extracts (30 μg) were separated by sodium dodecyl sulphate-polyacrylamide gel electrophoresis (SDS-PAGE) and the fractioned proteins were electro-transferred from the gel onto nitrocellulose membrane. The membrane was blocked overnight at 4ºC in a blocking buffer containing 10% (w/v) skimmed milk in PBS and 0.5% (v/v) Tween 20. The nitrocellulose membrane was washed three times with PBS containing 0.5% (v/v) Tween 20 (PBST) following incubation with primary antibodies anti-subtilisin polyclonal antibody (1:1500; 2 h at 25ºC), anti-cyspep antibody (1:10,000; overnight at 25ºC) and anti-calpain monoclonal antibody (1:5000; 2 h at 25ºC) in PBST (1:500). As a control, anti-β-actin polyclonal antibody produced in rabbit (Rhea Biotech) was used (1:5000; 2 h at 25ºC). After that, the membrane was washed three times with PBST and then incubated (1 h at 25ºC) with anti-rabbit-IgG-HRP (1:5000) for anti-subtilisin and anti-cyspep, and anti-mouse-IgG-HRP (1:5000) for anti-calpain. Following the additional three washing steps with PBST, bound antibodies were detected by chemiluminescence using SuperSignal West Pico Chemiluminescent Substrate. Gel Image were acquired and a semiquantitative analysis of bands density was performed using Fiji/ImageJ (version 1.54g) Gels Analysis Tool.

*Trypanothione reductase activity* - The samples were resuspended in 500 μL of lysis buffer^44^ and subjected to three freeze-thaw cycles. The soluble fraction was obtained by centrifugation (25,000 × g, 4ºC, 30 min) and the supernatant stored at -20ºC until further use. The protein concentration was determined using the Qubit™ Protein Assay Kit, following the manufacturer’s protocol. The enzymatic activity analysis was performed by a calorimetrical assay as previously described^44^ and modified by our group.^45^ The mix for the reaction (200 µL/well) was composed of 40 µg of intracellular amastigote protein or 8 μg of promastigote protein, as well as NADPH (200 µM), T[S]_2_ (30 µM to amastigote or 10 µM to promastigote) and DTNB (40 µM) in Tris-HCl buffer. The absorbance variation was measured at 412 nm for 50 min at 27ºC in a Molecular Devices SpectraMax spectrophotometer (Gemini XPS). The signal, in absorbance unit (AU), of reaction without T[S]_2_ (blank) was removed by subtraction of the total activity signal. The TR activity was calculated as variation of AU divided by time per mg of protein and expressed as AU × min^-1^ × mg^-1^ of protein.

*Assessing promastigote susceptibility to NaNO*_*2*_ *, H*_*2*_ *O*_*2*_ *and tert-butyl hydroperoxide* - Stationary phase promastigotes of *L. (V.) braziliensis* subpopulations Thor03, Thor10 and Thor22 and were seeded in 96-well plates (5.0 × 10^7^ per well) in Hank’s Balanced Salt Solution (HBSS) medium with NaNO_2_, H_2_O_2_ and tert-butyl hydroperoxide (tBOOH) solution in concentrations ranging from 32 mM to 0.5 mM, from 50 mM to 1.56 mM and from 41 mM to 1.28 mM, respectively.^10^ ^,^ ^31^ The compounds were incubated (4 h at 26ºC) along with 10 µL of resazurin prepared at 0.015%. The parasites survival was assessed by measuring resulting fluorescence variations using a SpectraMax M2 (Molecular Devices Inc.), with a 560 nm excitation and a 590 nm emission filter. The half-maximal effective concentration (EC50) value was calculated in GraphPad Prism version 8 (GraphPad Software, California USA, www.graphpad.com). Statistical analyses were performed among subpopulations for each compound. The one-way ANOVA was applied to compare the results, and data matrices were considered significantly distinct when the p-value was lower than 0.05.

*Ethical and law statements aspects* - Access to the protozoan *Leishmania* sp. was registered in the Brazilian System of Genetic Resource Management and Associated Traditional Knowledge (A41DBDD). Assays with animals were approved by the Committee for the Ethical Use of Animals of Instituto Oswaldo Cruz (CEUA-IOC: L0038/19).

## RESULTS

**Fig. 1:**
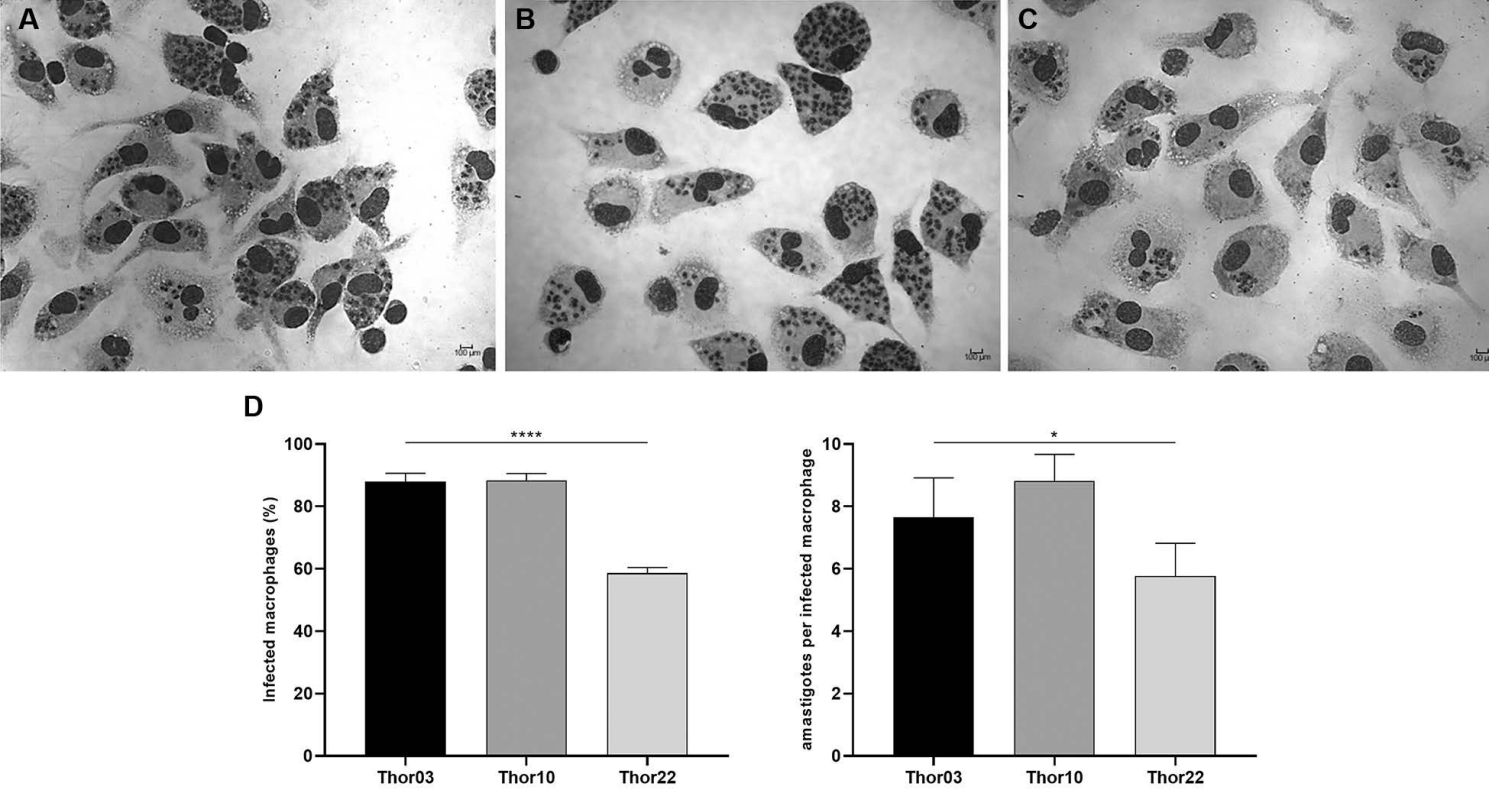
*in vitro* infection of peritoneal macrophages with subpopulations of *Leishmania (Viannia) braziliensis* Thor strain. Peritoneal macrophages (5 × 10^5^) were seeded and infected with metacyclic promastigotes (5 × 10^6^) from the subpopulations Thor03 (A), Thor10 (B) and Thor22 (C), at a ratio of 10:1 (parasite: macrophage). The infection was evaluated by calculating the percentage of infected cells and the number of parasites inside each infected cell (D). The figure shows images obtained from light microscopy after 24 h of parasite-macrophage interaction, stained using the Panoptic fast method. Scale bar: 100 μm. Data are representative of the mean and standard deviation of three independent experiments. To establish significant differences, the one-way analysis of variance (ANOVA) test was used * p = 0.0341 and **** p < 0.0001.

*Murine macrophage infection by subpopulations* - Since the subpopulations Thor03, Thor10 and Thor22 were kept cryopreserved in liquid nitrogen, it was necessary to evaluate the *in vitro* parasite infectivity, in which was assessed by tests with murine macrophages. To determine the percentage of infected cell and the number of parasites per macrophage, 100 cells were analysed per slide chamber. A comparable macrophages infection rate was observed for subpopulations Thor03 (88% ± 2.66%) and Thor10 (88.33% ± 2.22%), whereas Thor22 presented a lower infectivity rate (58.66% ± 1.77%) at 24 h of infection (Fig. 1). In contrast, the percentage of infected macrophages, the number of intracellular amastigotes per infected macrophage was not very distinctive among the tested subpopulations: Thor03 (7.66 ± 1.26), Thor10 (8.82 ± 0.85) and Thor22 (5.77 ± 1.05), (Fig. 1). As expected, the results confirmed the viability of the cryopreserved parasites, as well as their infectivity profiles, as described in previous publications.^37^ ^,^ ^38^

**Fig. 2:**
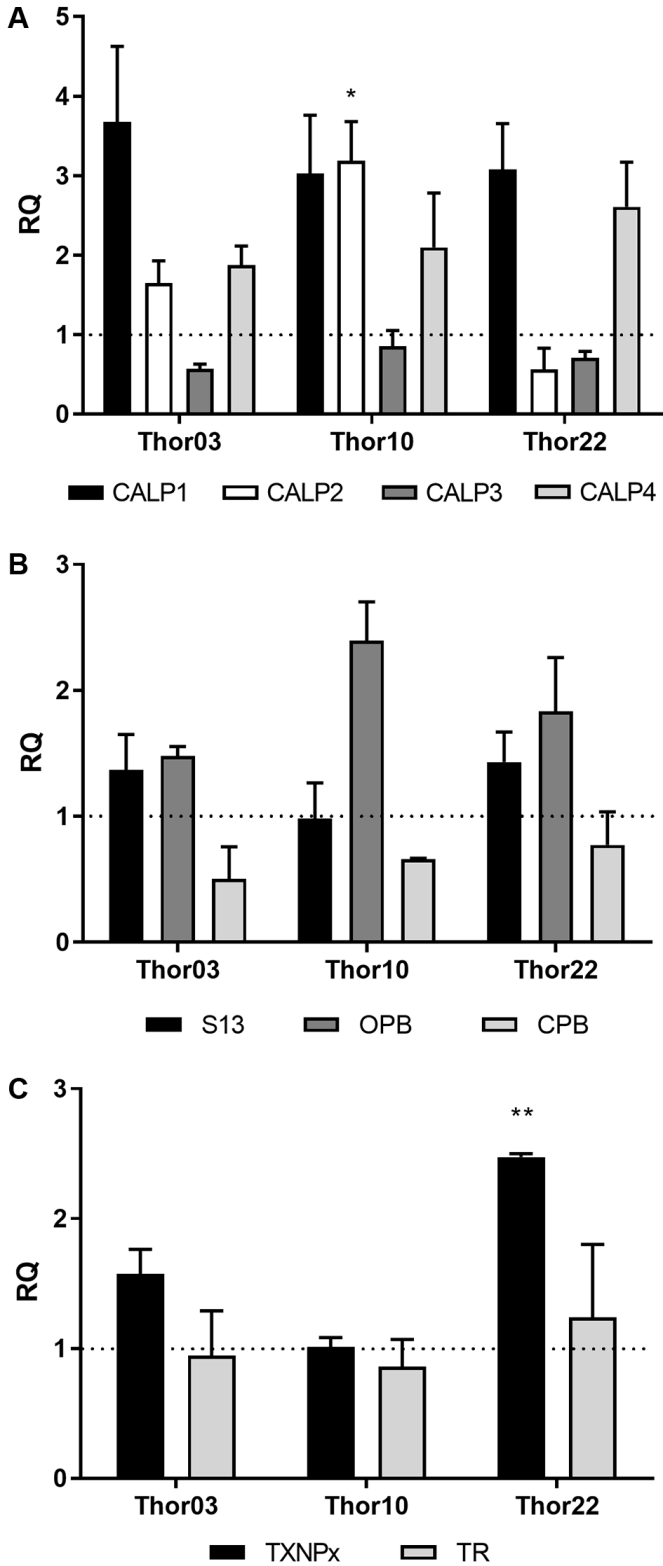
differential gene expression of Thor strain subpopulations. The representation of expression of calpain genes (A), as well as other cysteine protease (CPB) and serine proteases (subtilisin - S13 and oligopeptidase - OPB) (B), and the enzymes trypanothione reductase (TR) and tryparedoxin peroxidase (TXNPx) (C). Actin and ribosomal protein S8 were used as endogenous controls. The relative quantitation (RQ) values of intracellular amastigotes compared to promastigotes. The dashed line (= 1) indicates that the relative quantification level of intracellular amastigotes and promastigotes was equal. The graph presents the average of two independent experiments performed in triplicate. To establish significant differences between the RQ values of each gene, the one-way analysis of variance (ANOVA) test was used. * p = 0.0184 and ** p = 0.0085.

*Gene expression of protease and trypanothione system enzymes by RT-qPCR* - The expression of genes of cysteine proteases (calpains and CPB), serine proteases (S13 and OPB) and enzymes of the trypanothione system (TXNPx and TR) was evaluated in subpopulations of the Thor strain (Fig. 2). The highest RQ value observed was for the CALP1 gene, coding for calpain, in Thor03 (3.67 ± 1.34), whereas the lowest RQ value observed in the assays was for CPB gene in amastigotes of Thor03 (0.5 ± 0.36).

The results of the analysed subpopulations indicated higher expression levels of the gene CALP1 coding for calpain in the intracellular amastigote forms, followed by the genes CALP2, CALP4 and CALP3, with RQ values range from 0.57 ± 0.08 to 3.67 ± 1.34 in Thor03 (Fig. 2A). Only the assays performed for the CALP2 gene indicated RQ values with statistical significance (p = 0.0184) among the three subpopulations, with a higher expression rate in Thor10. Furthermore, this gene presented a trend of higher expression in promastigote forms of Thor22 (Fig. 2A).

The analysis of the expression of the S13 gene revealed similar RQ values between the three subpopulations (≤ 1.43 ± 0.23), a pattern which that was also observed for the CPB gene (≤ 0.77 ± 0.37) (Fig. 2B). Additionally, the expression of the OPB gene was detected in higher rates in Thor10 (2.39 ± 0.31) than in the other analysed subpopulations (Fig. 2B).

The expression of genes of trypanothione system enzymes (TXNPx and TR) showed values close to 1 for Thor03 and Thor10 (Fig. 2C). TR gene expression showed no significant differences among the subpopulations (Fig. 2C). The exception was the expression of the TXNPx gene in Thor22, which indicated an increasing in amastigotes with RQ values 2.47 ± 0.24, when compared to the other subpopulations (p = 0.0085).

**Fig. 3:**
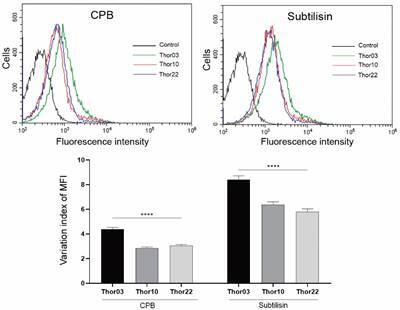
protease expression pattern in promastigotes. Flow cytometry histograms of Thor strain subpopulations (Thor03, Thor10 and Thor22) analysis using specific heterologous antibodies (anti-subtilisin and anti-cysteine protease B). Each histogram represents the analysis of 10,000 cells from one out of at least three experiments. Histogram colour: Thor03 (green line), Thor10 (red line), Thor22 (blue line), representative control (black line). The variation index of the mean fluorescent intensity (MFI) was obtained by dividing the MFI from labelled parasites by the non-stained autofluorescence control. To establish significant differences, the one-way analysis of variance (ANOVA) test was used. **** p < 0.0001.

*Protease expression levels by flow cytometry* - Flow cytometry was used to detect and quantify calpain, CPB and subtilisin proteases in the promastigotes from subpopulations Thor03, Thor10 and Thor22 (Fig. 3). The data generated in these assays was arranged in histograms and MFI values indicated that fluorescence intensity in the detection of subtilisin and CPB it was possible to detect differences among subpopulations. In this case, data of subtilisin showed the highest MFI values in Thor03 (8.40 ± 0.31), followed by Thor10 (6.37 ± 0.23) and Thor22 (5.81 ± 0.21); and for CPB as the highest fluorescence intensity in Thor03 (4.37 ± 0.16), followed by Thor22 (3.04 ± 0.11) and Thor10 (2.84 ± 0.10), (Fig. 3). These positivity rates for antigen and antibody reactions remained above those of control samples, promastigotes not marked by primary antibodies, also permeabilised with detergent and only incubated with secondary antibodies.

**Fig. 4:**
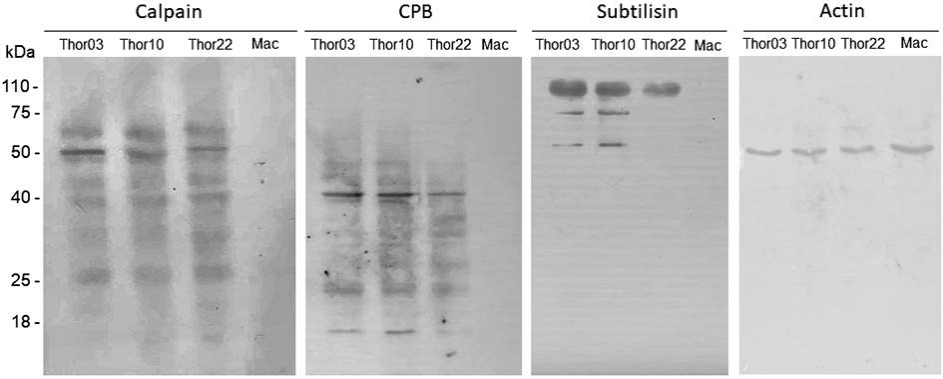
immunoblotting assays of proteases and actin of intracellular amastigotes. Protein samples (40 μg) from murine macrophages infected with subpopulations Thor03, Thor10, Thor22 or not infected (Mac) were applied into each slot, submitted to sodium dodecyl sulphate-polyacrylamide gel electrophoresis (SDS-PAGE), and transferred to a nitrocellulose membrane. After that, proteases (calpain, subtilisin and cysteine protease B) and actin were revealed by western blotting assays using specific antibodies and a Horseradish peroxidase (HRP) conjugated secondary antibody. Bands were visualised by chemiluminescence. The molecular mass markers are indicated on the left (kDa). These results are representative of four independent experiments.

*Protease profile in protein extracts from intracellular amastigotes by western blotting* - Immunoblotting assays were performed to verify the presence of calpain, CPB and S13 proteases in total protein extracts from intracellular amastigotes of the subpopulations Thor03, Thor10, and Thor22 (Fig. 4). Qualitative analysis bands in the gel revealed main protein profiles when samples were incubated with anti-subtilisin (110 kDa to 50 kDa), anti-CPB (40 kDa to 14 kDa) and calpain (60 kDa to 25 kDa).

Semiquantitative analysis by densitometry of these protein bands indicated different intensity profiles [Supplementary data (Tables II-III)]. Protein samples from macrophages infected with Thor03 and Thor10 showed bands with the strongest intensity for subtilisin (110 kDa, 75 kDa, and 50 kDa) and CPB (40 kDa and 14 kDa). The analysis of anti-calpain antibody indicated the presence of more intense bands in Thor03 around 50 kDa, in addition to the presence of other bands (60 kDa, 40 kDa and 25 kDa) with comparable intensities in the three subpopulations analysed.

Control protein samples, obtained from peritoneal macrophages infected with subpopulations Thor03, Thor10 and Thor22 or from uninfected macrophages (Fig. 4), indicated that β-actin expression levels (for the 50 kDa band) are stable amongst the samples [Fig. 4, Supplementary data (Table II)].

**Fig. 5:**
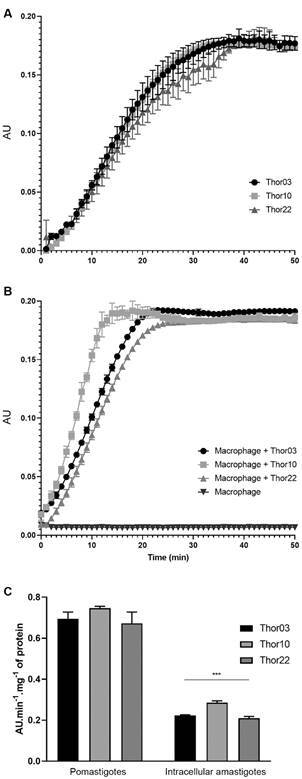
activity of trypanothione reductase (TR) in whole protein extracts of promastigotes and intracellular amastigotes from Thor strain subpopulations. Kinetic curves of TR activity of the subpopulations. Protein extract (8 μg) of Thor03, Thor10, Thor22 promastigotes (A) and (B) murine macrophages infected with these parasites were assessed. Data are presented as absorbance unit (AU) in time (min) and curves show the TR-mediated reduction of DTNB assessed during 50 min. The protein extract of non-infected macrophages also was assessed (B). The TR activity of promastigotes and intracellular amastigotes was calculated as variation of AU divided by time per mg of protein (C). Data are presented as the mean ± standard deviation of enzymatic activity (AU × min^-1^ × mg^-1^ of protein) of three independent experiments and the one-way analysis of variance (ANOVA) test used for statistical analysis. *** p = 0.0002.

*Trypanothione reductase activity in the protein extracts* - TR enzymatic activity was evaluated in total proteins extracts from promastigotes or from murine macrophages infected with amastigotes of the subpopulations Thor03, Thor10 or Thor22 (Fig. 5). In promastigote extratcs, the kinetic curves reached their plateau at around 40 min of reaction with values of approximately 0.18 AU (Fig. 5A), whereas in extracts of amastigote-infected macrophages, a plateau of 0.19 AU was reached at 15 min of reaction (for Thor10) or 20 min (for Thor03 and Thor22) (Fig. 5B). Protein extracts from uninfected macrophages did not present measurable TR activity (Fig. 5B).

TR enzymatic activity values were significantly higher in Thor10 (0.285 ± 0.006 AU × min^-1^ ×mg^-1^ of protein), followed by Thor03 (0.222 ± 0.002) and Thor22 (0.209 ± 0.007) (Fig. 5C). For protein extracts of promastigotes, the values were similar among the three subpopulations (Thor03 = 0.695 ± 0.02 AU × min^-1^ × mg^-1^ of protein; Thor10 = 0.746 ± 0.007; Thor22 = 0.672 ± 0.030), without statistical significance (Fig. 5C).

**Fig. 6:**
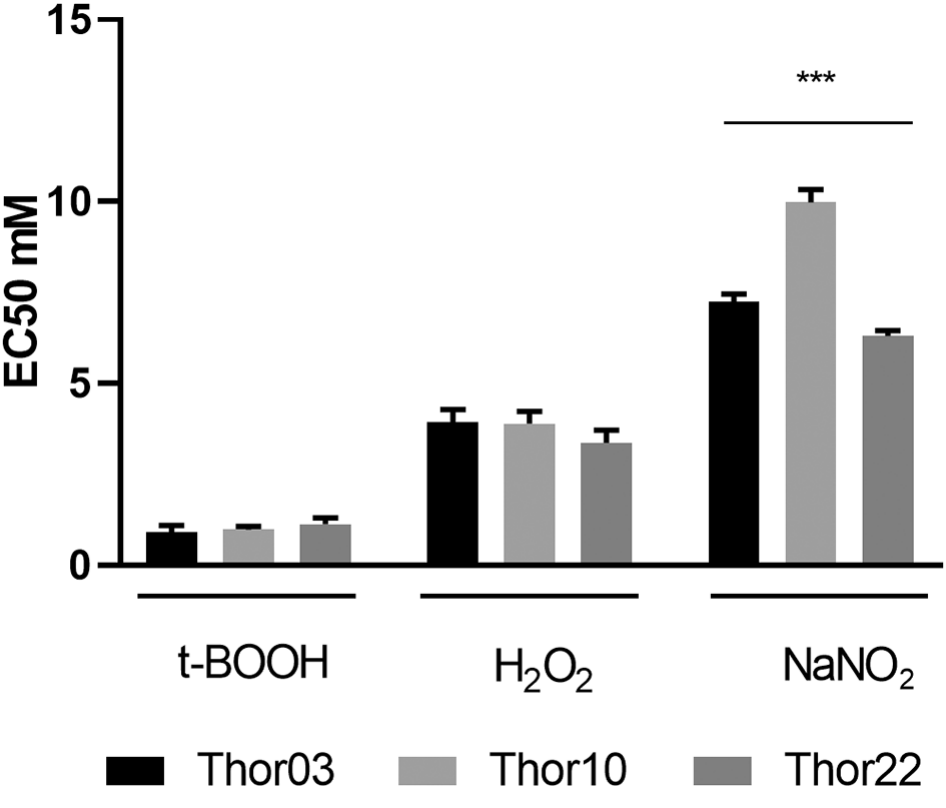
effect of incubating promastigotes with hydrogen peroxide-inducing reagents and nitric oxide (NO) donor. Promastigotes of subpopulations Thor03, Thor10, and Thor22 were seeded in the presence H_2_O_2_, tert-butyl hydroperoxide (tBOOH) and NaNO_2_ at different concentrations and the half-maximal inhibitory concentration in mM (EC50 mM) values were calculated. Data are presented as the mean ± standard deviation of three independent experiments. The one-way analysis of variance (ANOVA) was performed among subpopulations for each compound. *** p = 0.0001.

*Susceptibility of promastigotes to hydrogen peroxide-inducing agents and nitric oxide (NO) donor* - The effect of different concentrations of tBOOH, H_2_O_2_ or NaNO_2_ on promastigotes was examined as part of an effort to delineate variabilities in the susceptibility of the subpopulations to oxidative stress damage. In general, the data indicated higher EC50 values for NaNO_2_ (≥ 6.30 ± 0.22), followed by H_2_O_2_ (≥ 1.13 ± 0.49) and tBOOH (≥ 0.91 ± 0.18) (Fig. 6). The EC50 values of NaNO_2_ were significantly higher in Thor10 followed by Thor03 and Thor22 (Fig. 6). For tBOOH and H_2_O_2_, no differences in the susceptibility profiles of EC50 values were detected among the three analysed subpopulations (Fig. 6).

## DISCUSSION

Phenotypic diversity in *L. (V.) braziliensis* can be a key feature to its adaptive success. This issue has been explored through different approaches, contributing to a better understanding of how this parasite survives in an array of different environments during its life cycle, such as the digestive tract of the invertebrate host and the tissues and cells of the vertebrate host.^9^ Phenotypic characteristics including virulence factors and their final effects, such as the parasite’s oxidative stress response, were assessed for a better understanding of Thor strain subpopulations and their possible impacts on the host microenvironment.

In fact, cysteine proteases act at several steps of the parasite life cycle. The four calpain genes evaluated were previously assessed in *L. (V.) braziliensis* Thor strain and tended to be more expressed in metacyclic than procyclic promastigotes,^19^ therefore reinforcing the potential of these enzymes on driving the relationship with the host. In the present study, three of these genes were more expressed in intracellular amastigotes, being the CALP2 gene the most expressed in the Thor10 subpopulation. The exception was the CALP3 gene, which was more expressed in promastigotes for all analysed subpopulations. Furthermore, the dynamic expression of the protease genes points to different roles of these enzymes in each form of the parasite. The calpain genes expression pattern shows similar abundance of transcripts during infection, which was associated with a calpain protein profile. In western blotting assay, this profile was detected with the antibody specific to a consensus peptide sequence (LEKAYAKLHGSY) found in 13 calpain genes with predicted molecular masses ranging main from 100 to 53 kDa.^19^ It would be expected to observe multiple bands at various molecular weights in these assays. In fact, the profile detected here presented main bands between 75 KDa and 40 kDa, with presence of other bands below 31 kDa, as previous observed in *L. (V.) braziliensis* Thor strain.^19^ Therefore, this finding may be related to more than one action of these enzymes in the infection process by Thor strain subpopulations.

A dynamic correlation between cysteine protease gene expression and the respective protein was also assessed for a cathepsin of *L. (V.) braziliensis* in the Thor strain subpopulations. As it is known, CPB is a well-described virulence factor that shows multiple functions both in the modulation of immunological response of the mammalian host and in *Leishmania* sp. differentiation.^21^ ^,^ ^46^ Although the results obtained here indicate no significant expression of the genes that codes for CPB in amastigotes, the immunomodulatory role of this enzyme and respective COOH-terminal extension (cyspep), was noticed in *Leishmania* sp. as critical in repressing host immune response and therefore deletion of isoforms gene decreased parasite infection.^25^ ^,^ ^47^ This modulation has been related to the cyspep released during the maturation of pro-CPB,^40^ and could influence host immune response, changing the level of cytokines related to Th1 and Th2 responses.^48^ ^,^ ^49^ Additionally, cyspep fragment has shown efficacies in differentiating infected and uninfected dogs in areas endemic for leishmaniasis, based on the humoral immune response of these animals.^50^ In the macrophage infection, cyspep fragment is released during maturation of pro-CPB.^40^ In immunoblotting assays, as the antibody used is directed to this region, it is possible that this protein is being detected at different times during its processing. Thus, it is plausible to suggest that greater detection of bands close to 40kDa and 14kDa in Thor03 and Thor 10 could be associated to a greater ability to infect the host cell. Also, this finding could be related to the collaborative actions among these parasites to infection outcome since more infectious subpopulations (Thor10 and Thor03) provide a favourable environment for Thor22 infection,^38^ which in turn would have greater potential to interfere with modulation of the immune response of the vertebrate host.

Taking the multiple protease compositions and the respective functions in *Leishmania* spp., it was possible to explore serine protease as degradome components driving the *L. (V.) braziliensis* Thor strain subpopulation phenotype showing some particularities in the OPB and subtilisin genes expression. Both proteases’ classes are widely explored in *Leishmania* spp. studies.^51^ Data gathered here show an increased expression of OPB in amastigotes of assessed *L. (V.) braziliensis* Thor subpopulations, which is in accordance with the described for these enzymes as a molecular marker of this phase.^52^ In a previous study with *L. (V.) braziliensis* clinical isolates, OPB showed higher expression in intracellular amastigotes,^9^ which was also observed in the subpopulations highlighting the importance of this enzyme as a virulence factor. In addition, as OPB is not identified in human and other mammalian genome, this enzyme presents potential as a target for the development of drugs against leishmaniasis.^53^ ^,^ ^54^

Concerning subtilisin results, a similar expression of this gene was found in intracellular amastigotes of *L. (V.) braziliensis* clinical isolates with distincts macrophage infection profiles.^9^ Although the subpopulations showed no difference in gene expression, the immunoblotting assays showed a greater amount of the enzyme in promastigotes of Thor03, precisely one of the parasites with the highest infection rate. Regarding macrophage infection, data obtained by the specific recognition with anti-subtilisin antibody show a protein profile (110 kDa, 75 kDa and 50 kDa), as also shown in previous results using this antibody in promastigotes of *L. (L.) amazonensis* (144 kDa, 129 kDa and 57 kDa).^39^ Thus, based on these results it is possible to propose that this antibody detects degradation products of the same protein, whose native molecular weight is up to 110 kDa in both *Leishmania* spp. This protein profile was more intense in the infection samples from Thor03 and Thor10, which could be associated with the higher infection rate of these subpopulations.

Due to gathered data here and previously showed subtilisin role as a maturase of peroxidase enzyme of the trypanothione system^31^ and as a virulence factor,^8^ ^,^ ^9^ ^,^ ^41^ was opportune to explore molecular and biochemical approaches of redox system. In *Leishmania* spp., the trypanothione system plays a significant role in parasite protection against mammalian host defence systems by recycling disulfide to control the redox balance through the enzyme trypanothione reductase.^31^ ^,^ ^34^ Therefore, simulations of microenvironment conditions containing ROS and RNS-induced in the assays performed here were pivotal in confirming the resistance of promastigotes from Thor strain subpopulations since showing the highest value was for NaNO_2_ for Thor10, followed by Thor03 and Thor22. It is important to keep in mind that each parasite’s performance in the infection of macrophages is related to its evasion strategies of microbicidal responses. Therefore, the production of nitric oxide, hydrogen peroxide, and hydroperoxide (ROOH) are some of the macrophages defence mechanisms^55^ ^,^ ^56^ that were explored here to access the resistance of Thor subpopulation.

Based on these findings, additional concerns need to be commented on *L. (L.) infantum* isolates from patients that presented therapeutic failure were more resistant to NO and antimony *in vitro*, as well as showed the highest infection rates in macrophage infection assays.^57^ In the case of NO-resistant *L. (V.) braziliensis* parasites, they presented higher rates of survival and multiplication in macrophages than susceptible isolates.^10^ This resistance to nitric oxide has been correlated with more aggressive clinical forms observed in patients.^10^ ^,^ ^58^ The results of this study could explain the differences in the infection rate between the subpopulations once that difference in susceptibility to NO contributes to escape the macrophage microbicidal responses and enable survival and persistence of parasites. In addition, previous studies showed that strains of *L. (V.) braziliensis* less susceptible to NO presented higher infection rates and reduced levels of TNF-α production.^59^ ^,^ ^60^ In fact, it was noticed that lower NO and TNF-α production levels were observed for all infections with Thor10/Thor22 combinations.^38^

On the other hand, the effect of incubating promastigotes with hydrogen peroxide-inducing reagents and nitric oxide donors did not show significant variation to H_2_O_2_ and tBOOH between subpopulations. The U937 macrophage cells infected with *Leishmania (L.) chagasi* showed the role of ROS in controlling parasites at the beginning of the infection and the preponderant role of reactive RNS throughout the process.^56^ The antioxidant parasite defence against these RNS and ROS include thiols and specifically trypanothione system.^56^ *Leishmania* still maintains the glutathione (GSH) system, but this has less activity than trypanothione. Both antioxidant systems, glutathione and trypanothione, have roles in protection of parasites against the toxic effect of RNS.^61^ In addition, it was observed that T(SH)_2_ could be involved in the defence system against NO, since it complexes with NO greater affinity than GSH.^62^ Here, it was noticed lower TR activity in Thor22 amastigotes, while in promastigotes the activity was similar in the three subpopulations. This biochemical approach was previously used to evaluate clinical isolates of *L. (V.) braziliensis*, measuring the TR activity of promastigotes, axenic and intracellular amastigotes.^45^

In fact, the capacity of T(SH)_2_ to intercept NO was noticed in *in vitro* assays with *Leishmania (L.) infantum* promastigotes parasites exposed to NO.^62^ The downregulation of TR activity in *L. (L.) donovani* did not impact H_2_O_2_ processing in promastigotes, however but decreased the infection capacity of these parasites.^34^ While the overexpression of TR activity of *L. (L.) donovani* promastigotes not metabolised H_2_O_2_ at higher rates indicating that this enzyme is not a rate-limiting step in the H_2_O_2_ metabolism.^63^ In the case of peroxidase, overexpression in *Trypanosoma cruzi* and *L. (L.) infantum* increased resistance to exogenous H_2_O_2_ and tBOOH.^64^ ^,^ ^65^ Our findings showed that although gene expression assays did not show differences in TR levels, the enzymatic activity in intracellular amastigotes could explain the success of infection by the Thor10 subpopulation. These results could be related to polycistronic gene expression in *Leishmania* spp.^66^

In conclusion, evidence touching virulence factors by gene expression and protein detection tools used here to explore phenotype descriptors of subpopulations Thor03, Thor10 and Thor22 suggest a wide possibility of protease actions in *L. (V.) braziliensis* Thor strain. The data indicated a dynamic expression between the protease genes, suggesting different actions of these enzymes in both forms of the Thor strain subpopulations as descriptors of parasite virulence factors. The outcome caused by Thor subpopulation’s infectivity level is related to the property to resist redox state of macrophages.
