# Supplementary material for: Assessing proteases and enzymes of the trypanothione system in subpopulations of Leishmania (Viannia) braziliensis Thor strain during macrophage infection
Source: Mem Inst Oswaldo Cruz. 2024 Jul 8;119:e240038. doi: 10.1590/0074-02760240038 (PMC11251415; doi:10.1590/0074-02760240038)
Supplement: Supplementary file 1 [file 1678-8060-mioc-119-e240038-s.pdf]

TABLE I

Primers sequences and standard curve parameters for gene expression of *Leishmania (Viannia) braziliensis* Thor strain subpopulations

Gene target	Primer sequences	Reference	Amplicon length	Coefficient of linearity (R^2)	Amplification efficiency (%)
Subtilisin - S13 (LbrM.13.0860)	Fw ATC TGG CGA TTT CTC CCT TT<br>Rv GAG CTA ACA CCA G	[41]	201 bp	0.9958	92.52
OPB (LbrM.09.0850)	Fw GAG ACT CAG CGA CAG AGA AA<br>Rv ATA TCG TTT ATT CTT TCC CAC GC	[9]	122 bp	0.9917	109.71
CPB (LbrM.08.0810, LbrM.08.0820 and LbrM.08.0830)	Fw AAC CTG GAG CTG ATG CGC GA<br>Rv GGC GTC ACG GCG CCC TTC TC	This study	227 bp	0.9896	96.44
CALP1 (LbrM.18.1160)	Fw GGA GGC AGC TCA ACT CCG GT<br>Rv CAT TCC CAG CGC CAA CAC CC	[19]	106 bp	0.9993	87.98
CALP2 (LbrM.20.0290)	Fw TCG TCA GCG AAA CGG AGG GG<br>Rv ACG TAC TCT CGC GTG CCA GG	[19]	115 bp	0.9979	93.23
CALP3 (LbrM.20.5410)	Fw GCT TTC CCA CAT CGC GGC TG<br>Rv GTT GCC GGG GGT ACT CAG CA	[19]	134 bp	0.9999	95.56
CALP4 (LbrM.31.0600)	Fw TGG GTT ACG CTG GAG GCA GG<br>Rv AGG CCC ATC ACA TAC GGG CG	[19]	98 bp	0.9684	79.88
Tryparedoxin peroxidase (LbrM.15.1080)	Fw CTC TGT GGA CAG CGA GTA CG<br>Rv TGG GGT CGA TGA TAA AGA GG	[41]	166 bp	0.9991	81.43
Trypanothione reductase (LbrM.05.0350)	Fw GAAAAGGATGGCGAGGTGC<br>Rv AGATGCCTACGCTCTGAATGAT	[42]	76 bp	0.9952	71.78
40S ribosomal protein S8 (LbrM.24.2160)	Fw CGA CTT GGA TGC GGG GA<br>Rv GGC GAA GCC TTG TTC ACG	[41]	98 bp	0.9963	94.69
Actin (LbrM.04.1250)	Fw CAA GTG CGA CAT TGA TGT GCG<br>Rv ACG GCG CCA GGT TCG AGA T	[8]	119bp	0.9989	89.13

TABLE II

Semiquantitative analysis by densitometry using Fiji/ImageJ Gels Analysis Tool (Fiji Is Just ImageJ). Arbitrary unit (AU)

Protein	Molecular weight (kDa)	Thor03 (AU)	Thor10 (AU)	Thor22 (AU)
CPB	40	290	254	105
	14	62	85	20
Calpain	60	45	34	29
	50	91	38	33
	40	21	5	14
	25	26	26	29
Subtilisin	110	603	477	330
	75	72	132	0
	50	68	132	0
Actin	50	74	84	81

TABLE III

Relative density (RD): arbitrary unit (AU) of band / arbitrary unit (AU) of actin

Protein	Molecular weight (kDa)	Thor03 (RD)	Thor10 (RD)	Thor22 (RD)
CPB	40	3.92	3.02	1.29
	14	0.83	1.01	0.24
Calpain	60	0.61	0.40	0.36
	50	1.22	0.45	33
	40	0.28	0.06	0.40
	25	0.35	0.31	0.36
Subtilisin	110	8.14	5.68	4.07
	75	0.97	1.57	0
	50	0.92	1.57	0
Actin	50	1	1	1
